# Comparative analysis of CAMS aerosol optical depth data and AERONET observations in the Eastern Mediterranean over 19 years

**DOI:** 10.1007/s11356-024-32950-6

**Published:** 2024-03-19

**Authors:** Gizem Tuna Tuygun, Tolga Elbir

**Affiliations:** Department of Environmental Engineering, Faculty of Engineering, Dokuz Eylul University, Buca-Izmir, Turkey

**Keywords:** Aerosol optical depth (AOD), CAMS, Validation, AERONET, Eastern Mediterranean

## Abstract

Aerosol optical depth (AOD) is an essential metric for evaluating the atmospheric aerosol load and its impacts on climate, air quality, and public health. In this study, the AOD data from the Copernicus Atmosphere Monitoring Service (CAMS) were validated against ground-based measurements from the Aerosol Robotic Network (AERONET) throughout the Eastern Mediterranean, a region characterized by diverse aerosol types and sources. A comparative analysis was performed on 3-hourly CAMS AOD values at 550 nm against observations from 20 AERONET stations across Cyprus, Greece, Israel, Egypt, and Turkey from 2003 to 2021. The CAMS AOD data exhibited a good overall agreement with AERONET AOD data, demonstrated by a Pearson correlation coefficient of 0.77, a mean absolute error (MAE) of 0.08, and a root mean square error (RMSE) of 0.11. Nonetheless, spatial and temporal variations were observed in the CAMS AOD data performance, with site-specific correlation coefficients ranging from 0.57 to 0.85, the lowest correlations occurring in Egypt and the highest in Greece. An underestimation of CAMS AOD was noted at inland sites with high AOD levels, while a better agreement was observed at coastal sites with lower AOD levels. The diurnal variation analysis indicated improved CAMS reanalysis performance during the afternoon and evening hours. Seasonally, CAMS reanalysis showed better agreement with AERONET AODs in spring and autumn, with lower correlation coefficients noted in summer and winter. This study marks the first comprehensive validation of CAMS AOD performance in the Eastern Mediterranean, offering significant enhancements for regional air quality and climate modeling, and underscores the essential role of consistent validation in refining aerosol estimations within this complex and dynamic geographic setting.

**Supplementary Information:**

The online version contains supplementary material available at 10.1007/s11356-024-32950-6.

## Introduction

Atmospheric aerosols, comprising tiny particles such as dust, smoke, and other pollutants suspended in the air, significantly impact air quality, human health, and the environment (Pöschl 2005; McNeill 2017). Aerosol optical depth (AOD) measures how much sunlight is absorbed or scattered by these atmospheric aerosols. As a crucial parameter for characterizing the atmosphere’s optical properties, AOD provides insights into the total aerosol content and its size, shape, and composition (Wei et al. 2020; Vogel et al. 2022). By assessing AOD, it is possible to determine the impact of atmospheric aerosols on air quality, human health, and climate change (Tuna Tuygun et al. 2021; Gündoğdu et al. 2022; Qi et al. 2022; Tuna Tuygun and Elbir 2023a).

AOD data can be sourced from various platforms, including (i) ground-based monitoring stations equipped with sun photometers, sky radiometers, and ceilometers, which measure direct and scattered sunlight to ascertain atmospheric aerosol concentration (Kim et al. 2008; Khor et al. 2014); (ii) satellite-based instruments such as the Moderate Resolution Imaging Spectroradiometer (MODIS), Multi-angle Imaging SpectroRadiometer (MISR), and Polarization and Anisotropy of Reflectances for Atmospheric Sciences coupled with Observations from a Lidar (PARASOL), which determine aerosol concentration by measuring direct and scattered sunlight (Tuna Tuygun et al. 2020; Chiapello et al. 2021; Gui et al. 2021); (iii) numerical models like the Global Modeling and Assimilation Office (GMAO) and the Community Atmosphere Model (CAM), which simulate atmospheric conditions and use data from various sources, including satellite and ground-based measurements, to estimate aerosol concentration (Bacmeister et al. 2014; Gelaro et al. 2017); and (iv) aerosol data assimilation systems such as the Copernicus Atmosphere Monitoring Service (CAMS) and the European Space Agency’s (ESA) Climate Change Initiative (CCI) project, which integrate satellite-based measurements, ground-based observations, and numerical models to produce comprehensive aerosol data (Peuch et al. 2022).

The CAMS aerosol service, a component of the European Union’s Copernicus Earth Observation program, offers near real-time data on atmospheric aerosol levels and distribution across Europe and adjacent regions. This service combines satellite data, ground-based observations, and numerical models to generate high-resolution AOD and other aerosol data. CAMS products support a wide array of applications, including air quality monitoring and forecasting, climate research, and evaluating atmospheric aerosols’ impacts on human health and the environment, by providing timely and accurate information essential for decision-making by public health authorities, environmental agencies, and other stakeholders (Casciaro et al. 2022; Sbai et al. 2022).

Recent global and independent studies have begun to validate CAMS aerosol data (Gueymard and Yang 2020; Misra et al. 2020; Ukhov et al. 2020; Witthuhn et al. 2020; Zhang et al. 2020; Isaza et al. 2021; Salamalikis et al. 2021; Garrigues et al. 2022). CAMS also released a report detailing the global validation results of its aerosol and reactive trace gas reanalyses from 2003 to 2021 (Kapsomenakis et al. 2022). Although the study by Gueymard and Yang (2020) assessed the regional performance of CAMS AOD products, it identified a lack of test stations in certain areas. Nevertheless, it concluded that CAMS AOD products generally provide accurate and consistent AOD estimates, demonstrating acceptable agreement with ground- or satellite-based measurements. However, studies in the Eastern Mediterranean region have predominantly used the Modern-Era Retrospective Analysis for Research and Applications, Version 2 (MERRA-2) data for aerosol research instead of CAMS data (Aldabash et al. 2020; Shaheen et al. 2020, 2021). Despite MERRA-2 assimilating data from more observation sources than CAMS, their spatial representation and consistency can significantly vary (Buchard et al. 2017). Thus, the representativeness of reanalysis data in different regions warrants examination, as it can be influenced by several factors, including the assimilation model physics, resolution, and limitations in the data sources used for assimilation.

Given these considerations, it is valuable to investigate the representativeness of CAMS AOD on a continental scale and assess the potential impact of assimilated aerosol information on reanalysis data. Notably, independent validation of CAMS AOD in the Eastern Mediterranean region has not yet been conducted. This study aims to evaluate CAMS AOD accuracy in the region to understand the causes of bias or random errors in AOD retrieval across various spatial and temporal scales. Although this study does not specifically address the impact of database accuracies on derived particulate matter estimates at the regional level, this aspect can be explored in subsequent research contributions.

AERONET, a ground-based aerosol monitoring network established by NASA, provides optical and microphysical properties of aerosols based on direct sun and diffuse sky measurements in different spectral bands (Holben et al. 1998). AERONET’s AOD measurements, often used as reference data for validating satellite-based aerosol observations and atmospheric models, were employed in this study to evaluate CAMS AOD data performance over the Eastern Mediterranean region using 19 years of observations. This study aims to assess CAMS AOD data precision, identify any limitations, and offer a comprehensive understanding of the spatiotemporal aerosol distribution in the region. Additionally, it examines the influence of various factors, including diurnal variation, seasonal fluctuations, and desert dust transport, on CAMS AOD data precision. The results of this study could guide the usage of CAMS AOD products for various applications in the Eastern Mediterranean region.

## Study area

The Eastern Mediterranean Region, strategically positioned at the crossroads of Europe, Asia, and Africa, is one of the world’s regions richest in geological and climatological diversity. The region covers an area of about 2.5 million km^2^, extending from the Balkan Peninsula in the north to the Arabian Peninsula in the south and from the Aegean Sea in the west to the Persian Gulf in the east. It is characterized by a semi-arid climate, with hot summers, mild winters, and an average annual temperature range of 10 °C to 25 °C. Precipitation varies widely based on location and elevation, from 100 to 1000 mm annually. Atmospheric circulation patterns influencing the area include Mediterranean cyclones, the Siberian anticyclone, the North Atlantic Oscillation, and the African monsoon, each contributing to the region’s distinct climate dynamics (Lelieveld et al. 2002; Rubin et al. 2007; Kourtidis et al. 2015).

Prominent geological features of the Eastern Mediterranean include the Taurus Mountains in Turkey and the Anti-Lebanon Mountains in Syria and Lebanon, which, along with coastal plains and plateaus, foster diverse ecosystems such as forests, deserts, and semi-arid scrublands. This biodiversity hotspot is home to over 10,000 plants and 1000 animal species, many of which are endemic or endangered (Quezel and Barbero 1982; Médail and Quézel 1999; Myers et al. 2000). The region’s geological activity is underscored by a complex network of fault lines and several active and dormant volcanoes, highlighted by significant geological features like the Dead Sea Transform fault system and the Afro-Arabian rift system. These structures have led to the formation of unique landscapes such as the Dead Sea, the Earth’s lowest point, and the Red Sea, known for its high salinity and biological diversity (le Pichon et al. 2019; Merry et al. 2021; Ben-Avraham et al. 2008; Charrach 2019).

Various natural and anthropogenic sources influence the atmospheric aerosol composition in the Eastern Mediterranean (Kaskaoutis et al. 2011; Kallos et al. 2014; Liakakou et al. 2020; Ozdemir et al. 2020; Tuna Tuygun and Elbir 2023b). The primary sources of atmospheric aerosols are desert dust, sea salt, and anthropogenic emissions from industrial activities and transportation (Elbir 2002; Kaskaoutis et al. 2008; Elbir et al. 2010; Kara et al. 2014, 2015; Tuygun et al. 2017; Ozdemir et al. 2020). Desert dust can be transported long distances from the Sahara Desert and Arabian Peninsula, affecting air quality in the Eastern Mediterranean region (Kaskaoutis et al. 2008; Gkikas et al. 2013; Varga et al. 2014; Bodenheimer et al. 2019). Sea salt is generated by wave breaking and sea spray, especially during strong winds and storms, contributing to the coarse mode of aerosols (Sellegri et al. 2001; Sciare et al. 2010). Anthropogenic aerosols are also significant contributors to the atmospheric aerosol composition in the region (Piazzola et al. 2012). In major urban areas such as Istanbul, Ankara, Athens, and Cairo, emissions from transportation, energy production, and industrial activities result in high levels of these aerosols (Elbir et al. 2010; Kanakidou et al. 2011; Kara et al. 2014).

Additionally, forest fires, agricultural, and biomass burning are notable sources of atmospheric aerosols, further contributing to the region’s complex and dynamic aerosol composition (Kaskaoutis et al. 2011; Bougiatioti et al. 2014; Garcia-Hurtado et al. 2014). Understanding these diverse sources in the region is essential for improving air quality and mitigating the impacts of air pollution on human health.

## Data and method

### CAMS reanalysis data

CAMS is integral to the European Union’s Copernicus Earth observation program, providing comprehensive environmental data derived from modeling, satellite observations, and in-situ measurements. CAMS employs the Integrated Forecasting System (IFS) developed by the European Centre for Medium-Range Weather Forecasts (ECMWF). In its assimilation process, the system incorporates aerosol data from the Advanced Along-Track Scanning Radiometer (AATSR) on board the Envisat satellite from 2003 to 2012 and data from the MODIS instrument on NASA’s Terra and Aqua satellites, which have been in operation from 2003 to the present. CAMS does not apply a bias-adjustment algorithm in its data assimilation and does not include ground-based AOD measurements. This exclusion of ground-based data renders AERONET a particularly suitable and independent reference for validating CAMS data.

This study retrieved 3-hourly AOD values at a wavelength of 550 nm from the CAMS reanalysis database, covering 19 years from January, 2003, to December, 2021. The data, at a 1° spatial resolution, were acquired through the Copernicus Atmosphere Data Store (https://ads.atmosphere.copernicus.eu/) utilizing a Python script, with detailed access instructions on the website. The CAMS AOD data for the Eastern Mediterranean region were subsequently validated against ground-based AOD measurements from 20 AERONET sunphotometer stations.

### AERONET data

The AERONET, a comprehensive ground-based network of sun photometers, measures aerosol optical and microphysical properties. These measurements, ranging between 340 and 1020 nm across multiple wavelengths, utilize direct sun and diffuse sky radiations. Due to their high accuracy, quality, and availability, AERONET data are extensively used as the reference standard for validating satellite-derived and model-based AOD products (Holben et al. 1998).

**Fig. 1:**
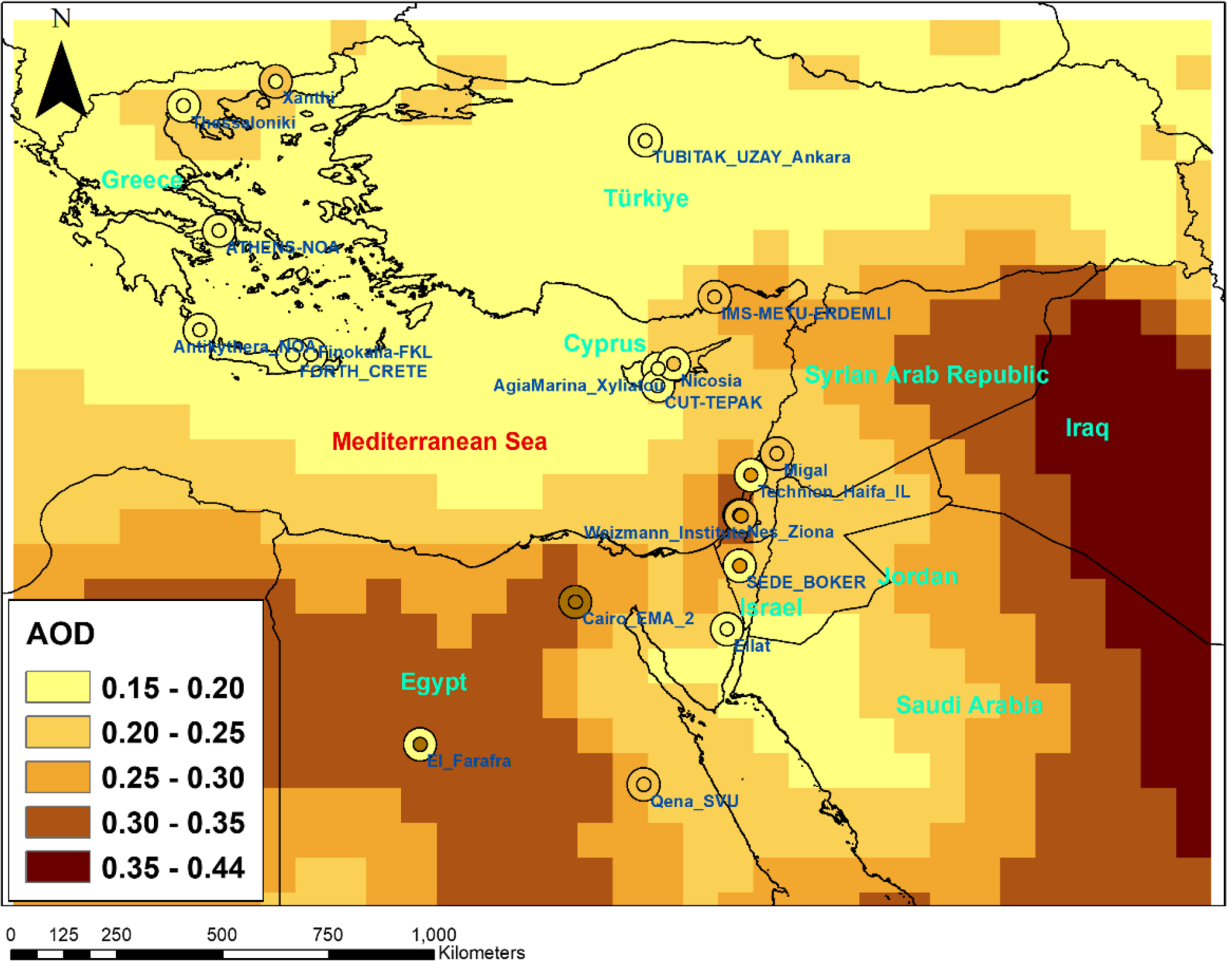
Spatial distribution of CAMS AOD across the Eastern Mediterranean (2003–2021) and corresponding AERONET stations

The recently released Version 3 AERONET Level 2.0 dataset was employed in the present study, specifically utilizing AOD measurements at 500 nm from 20 stations. Level 2.0 data, subject to quality assurance and cloud screening, can be accessed at https://AERONET.gsfc.nasa.gov/. Holben et al. (1998) provide comprehensive details regarding the AERONET network. Selection criteria for the stations included data availability for the study period, spanning from 2003 to 2021. Figure 1 presents the Eastern Mediterranean Region and the AERONET stations included in this study. The coordinates of these stations are also detailed in Table 1.

**Table 1:** Characteristics and AOD descriptive statistics for AERONET stations

Station name	Country	Latitude (N°)	Longitude (E°)	Data Availability	AOD at 550 nm	
					Mean ± std dev	Min–max
CUT-TEPAK	Cyprus	34.675	33.043	2010–2021	0.166 ± 0.103	0.017–1.468
AgiaMarina_Xyliatou	Cyprus	35.038	33.058	2015–2021	0.142 ± 0.102	0.012–1.064
Nicosia	Cyprus	35.141	33.381	2015–2021	0.166 ± 0.093	0.02–1.338
Cairo_EMA_2	Egypt	30.081	31.290	2010–2019	0.338 ± 0.184	0.045–3.879
El_Farafra	Egypt	27.058	27.990	2014–2018	0.173 ± 0.173	0.013–1.76
Qena_SVU	Egypt	26.200	32.747	2018–2019	0.209 ± 0.129	0.024–1.227
ATHENS–NOA	Greece	37.972	23.718	2008–2018	0.165 ± 0.098	0.008–1.137
Antikythera_NOA	Greece	35.861	23.310	2018–2020	0.143 ± 0.081	0.029–0.75
Finokalia–FKL	Greece	35.338	25.670	2014–2020	0.14 ± 0.097	0.005–0.935
FORTH_CRETE	Greece	35.333	25.282	2003–2017	0.167 ± 0.099	0.007–1.424
Thessaloniki	Greece	40.630	22.960	2003–2021	0.186 ± 0.113	0.006–1.404
Xanthi	Greece	41.147	24.919	2008–2015	0.217 ± 0.125	0.017–1.401
Eilat	Israel	29.503	34.517	2007–2020	0.185 ± 0.139	0.011–4.106
Migal	Israel	33.236	35.578	2018–2021	0.208 ± 0.126	0.024–2.159
Nes_Ziona	Israel	31.922	34.789	2000–2015	0.206 ± 0.144	0.015–3.661
SEDE_BOKER	Israel	30.855	34.782	1995–2020	0.163 ± 0.116	0.002–3.284
Technion_Haifa_IL	Israel	32.776	35.025	2016–2019	0.174 ± 0.111	0.02–1.122
Weizmann_Institute	Israel	31.907	34.811	2015–2020	0.192 ± 0.115	0.013–1.308
IMS–METU–ERDEMLI	Turkey	36.565	34.255	1999–2019	0.219 ± 0.133	0.014–1.848
TUBITAK_UZAY_Ankara	Turkey	39.891	32.778	2009–2017	0.175 ± 0.086	0.029–1.033

It was observed that only the Thessaloniki station provided a complete dataset throughout the study period. The descriptive statistics of AOD across the region revealed variability, with mean AOD values ranging from 0.140 to 0.338. Higher AOD values were predominantly recorded in southern countries like Egypt and Israel. Notably, the maximum 15-min AOD values reported were 4.106 at the Eliat station, 3.879 at the Cairo_EMA_2 station in Egypt, and 3.661 at the Nes_Ziona station in Israel. Conversely, lower AOD values were more frequently recorded in the northern countries, such as Greece and Turkey. Nevertheless, all stations experienced days with 15-min AOD values surpassing 0.7, especially during the spring, with the Cyprus stations occasionally recording peak levels up to 3.5. These observations underscore the significant aerosol load prevalent in the region.

Regarding the dataset in Table 1, Antikythera_NOA reported the fewest collocated data points (*N* = 841) since it was established in 2018 at the National Observatory of Athens in Greece. In contrast, another Greek station, the Thessaloniki station, reported the highest number of collocated data points (*N* = 19,169) from 2003 to 2021.

### Evaluation method

CAMS and AERONET AOD comparisons were conducted at the 20 AERONET sites depicted in Fig. 1. In these comparisons, AERONET data were assumed to reflect the actual AOD. It was imperative to undertake spatiotemporal matching to address the differences in spatiotemporal sampling between the AERONET and CAMS datasets. A standard methodology involving a 60-min temporal window and a 0.55° spatial window was employed (Ichoku et al. 2002; Sayer et al. 2014). To accommodate the temporal resolution of the CAMS reanalysis data, irregular 15-min AERONET measurements were aggregated into 3-h averages using Rstudio version 3.5.2. Matching was restricted to the availability of AERONET data during daylight hours, adjusted for Local Solar Time (LST), with the condition that AERONET observations be considered valid if comprising at least three instantaneous values.

Given the CAMS AOD outputs are reported at 550 nm, while AERONET provides AOD at either 440 nm or 500 nm, interpolation of AERONET AOD to 550 nm was necessary, employing the Ångström power law as described in the following equation:

1 $${{\text{AOD}}}_{550}={{\text{AOD}}}_{{\text{WL}}}\times {\text{exp}}\left[- {\alpha }_{{\text{AER}}} {\text{In}}\left(550/WL\right)\right]$$

Here, WL indicates the original wavelength (440 or 500 nm), while *α*_AER_ represents the Ångström exponent derived from AERONET measurements at 440 nm and 675 nm wavelengths. The selection of AOD data from 11 stations was due to their higher availability at the 500-nm wavelength, contrasting with nine stations providing data at 440 nm.

The study employed several evaluation metrics to assess the comparisons: (1) the count (*N*) of matched points, (2) the Pearson correlation coefficient (*R*) to evaluate the consistency of variations between measurements and retrievals, (3) the root mean square error (RMSE) as an indicator of an overall discrepancy, and (4) the mean absolute error (MAE) to measure the extent of actual retrieval errors.

## Results

### Spatial and temporal variation of CAMS AOD

Figure 1 provides a comprehensive visualization of the spatial distribution of average CAMS AOD values across the Eastern Mediterranean from 2003 to 2021. The AOD levels on the map facilitate an analysis of aerosol variations across different regions. The figure uniquely incorporates dual-colored circles at AERONET station locations, with the inner color representing the average CAMS AOD and the outer color indicating the average AERONET AOD for the corresponding period. This dual representation allows for a direct comparison between satellite-derived and ground-based AOD measurements, highlighting areas of unity and divergence.

Regions depicted with higher CAMS AOD ranges, particularly those exceeding 0.35, align with areas known for significant aerosol emissions, both natural, such as desert dust, and anthropogenic, from urban pollution and industrial activities. In contrast, regions with AOD values in the lower ranges suggest clearer atmospheric conditions, potentially indicative of lesser anthropogenic impact and lower natural aerosol contributions.

A closer examination of the AERONET stations, as detailed in Table 1, reveals variability in mean AOD values, with stations like Cairo_EMA_2 in Egypt showing higher mean CAMS AOD reflective of the dense aerosol presence, likely due to both natural (desert dust) and anthropogenic sources. Conversely, stations in Cyprus and certain areas of Greece report lower mean AOD values, suggesting clearer conditions in these parts of the region.

The comparison between CAMS and AERONET data at individual station locations offers insight into the model’s performance in capturing spatial aerosol distributions. Stations with closely matched CAMS and AERONET AOD colors indicate areas where the CAMS model accurately represents aerosol concentrations. In contrast, discrepancies in color between the inner and outer circles highlight regions where further investigation may be needed to understand the causes of divergence between modeled and observed data. The agreement between CAMS and AERONET indicates that highly close values are obtained in the northern regions, but there are large differences in the southern regions. This situation suggests that the CAMS reanalysis may underestimate the AOD in areas with high aerosol load, such as desert dust and urban pollution.

**Fig. 2:**
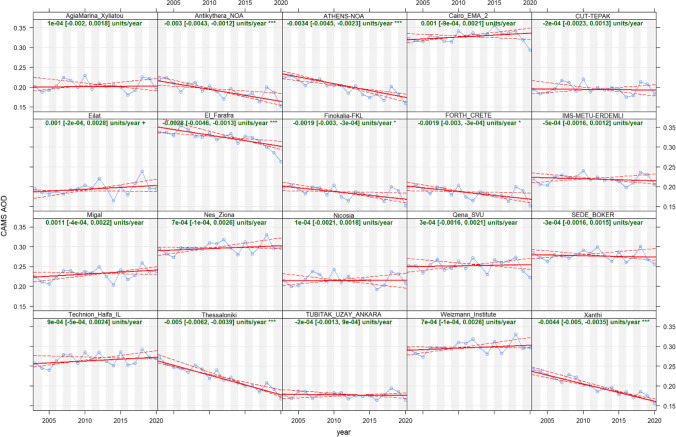
Temporal trends of CAMS AOD at AERONET stations

Figure 2 presents the temporal trend analysis of CAMS AOD at AERONET station locations within the Eastern Mediterranean Region from 2003 to 2021, employing the Theil-Sen estimator, which is a robust non-parametric method suitable for estimating linear trends in datasets with significant variability (Theil 1950; Sen 1968). Most stations exhibit a negative trend, suggesting a general decrease in aerosol loading over the analyzed period. Conversely, some stations demonstrate a positive trend, indicating increased aerosol loading in specific locales. Significant trends at the 0.001 level underscore high confidence in these estimates at certain stations. In contrast, other stations show non-significant trends, reflecting lower confidence levels in the trend estimates and potentially more stable aerosol conditions over time.

In examining the temporal trends of CAMS AOD data, Fig. 2 reveals distinct patterns when categorized by geographical locations. The stations in the northern regions, encompassing parts of Greece, Cyprus, and Turkey, display a slight downward or stable AOD trend over the study period. This situation suggests a relative constancy in aerosol levels, potentially reflective of steady climatic conditions or the efficacy of regional aerosol management strategies.

Conversely, the southern stations, particularly in Egypt and southern Israel, exhibit more pronounced variability. Notably, the Cairo_EMA_2 station demonstrates a trend of increasing AOD, possibly indicative of escalating urban and industrial emissions or the influence of desert dust transport from nearby arid zones. The arid and semi-arid regions of Egypt and Libya are known to be affected by thermal contrasts between land masses and surrounding waters, which can intensify aerosol mobilization from desert dust sources (Liakakou et al. 2020).

When differentiating between coastal and inland stations, the former typically register lower AOD values, with some fluctuations that may be linked to maritime influences or regional policy implementations. Inland stations, especially those proximate to dense urban centers or industrial hubs, show a more marked upward trend in AOD, underscoring the impact of anthropogenic factors. Coastal areas, particularly in Turkey, Greece, Cyprus, and Israel, experience sea-land breeze circulations that can amplify aerosol emissions from both marine sources and urban activities (Sciare et al. 2008).

A comparison between eastern and western stations further illustrates the influence of geography on AOD trends. Western stations, situated closer to the Mediterranean coast, often experience less variation in AOD, potentially due to maritime air flows and stringent emission regulations within the European Union. In contrast, eastern stations, lying closer to desert regions, exhibit greater AOD variability, likely due to both natural desert dust influences and variable emission controls. Mountainous regions, such as those in Lebanon, Syria, and Jordan, are subject to orographic effects that modulate aerosol vertical transport (Kallos et al. 2014).

Additionally, to complement the analysis of temporal trends in CAMS AOD presented in Fig. 2, a supplementary figure (Fig. S1) has been provided, illustrating the spatial variation of CAMS AOD across the Eastern Mediterranean in 5-year intervals. This figure, included in the Supplementary Material, offers a more granular view of the changes in aerosol loading over distinct periods, capturing the evolution of aerosol distribution patterns in the region.

These geographical categorizations of AOD trends underscore the complex interplay between natural and anthropogenic factors in shaping regional aerosol distributions. The data suggests that coastal and northern areas of the Eastern Mediterranean benefit from relatively better air quality conditions compared to their southern and inland counterparts, where AOD levels are more susceptible to variability and are influenced by a mix of factors, including urbanization, industrial activity, and natural desert dust dynamics.

### Validation of CAMS AOD against AERONET observations

An intercomparison study was conducted to assess the accuracy and spatial variability of CAMS AOD using 19 years of AERONET observations over the Eastern Mediterranean region. CAMS AOD was validated at 20 AERONET sites, encompassing a total of 112,703 matchups. The overall *R* of 0.7, along with an RMSE of 0.11 and an MAE of 0.08, indicates a substantial correlation between CAMS data and ground-based observations in the region (see Supplementary Material, Fig. S2). Notably, in Fig. S2, the regression line’s slope is less steep than the 1:1 line at higher AOD levels, suggesting a systematic underestimation of AOD by CAMS, particularly in regions with significant dust activity such as the Sahel and the broader Mediterranean, as discussed in Schulz et al. (2020) and Garrigues et al. (2022).

It is posited that the underestimation may, in part, be due to CAMS’ aerosol modeling system, which assimilates data from five aerosol types, excluding nitrates, an omission noted by Zhang et al. (2020) to potentially contribute to discrepancies in regions with high nitrate fractions like Europe and China. This underestimation is consistent with findings from various studies evaluating CAMS AOD products against AERONET data in different areas (Isaza et al. 2021; Ukhov et al. 2020; Lee et al. 2021; Salamalikis et al. 2021), such as the correlation range of 0.65 to 0.87 reported by Ukhov et al. (2020) in the Middle East. Kapsomenakis et al. (2022) also noted that the CAMS reanalysis tends to underestimate AOD compared to ground-based observations, especially in desert regions where mineral dust aerosols predominate.

For AOD values less than 0.5, which typically represent cleaner atmospheric conditions with lower aerosol loading, the scatter plot in Fig. S2 shows a higher density of points closer to the 1:1 line, indicating a better agreement between CAMS and AERONET data, corroborating results from Zhang et al. (2020). The *R*-value in this range is robust, which implies that CAMS AOD estimations tend to be more reliable in conditions of lower aerosol concentrations.

As AOD values rise above 0.5, indicating more polluted conditions with higher aerosol content, the validation performance exhibits some divergence from the 1:1 line. This situation suggests that CAMS AOD estimations may not capture the aerosol load as accurately in such conditions. The potential reasons for this discrepancy might include the increased complexity of aerosol types present in higher concentrations, challenges in satellite retrieval algorithms under heavy aerosol loading, and limitations in the model’s ability to simulate such conditions accurately. CAMS AOD’s weaker performance at higher AOD levels may be attributed to the presence of varied aerosol types and sources, including dust, sea salt, biomass burning, and anthropogenic pollutants in the region (Kaskaoutis et al. 2008; Gkikas et al. 2013; Bougiatioti et al. 2014; Ozdemir et al. 2020). These aerosols’ diverse optical and physical properties can challenge the accuracy of CAMS AOD retrievals (Dubovik et al. 2002; Mishchenko et al. 2004; Eck et al. 2010). Additionally, complex atmospheric conditions at higher AOD levels, such as cloud cover and atmospheric mixing, could influence retrieval accuracy.

**Fig. 3:**
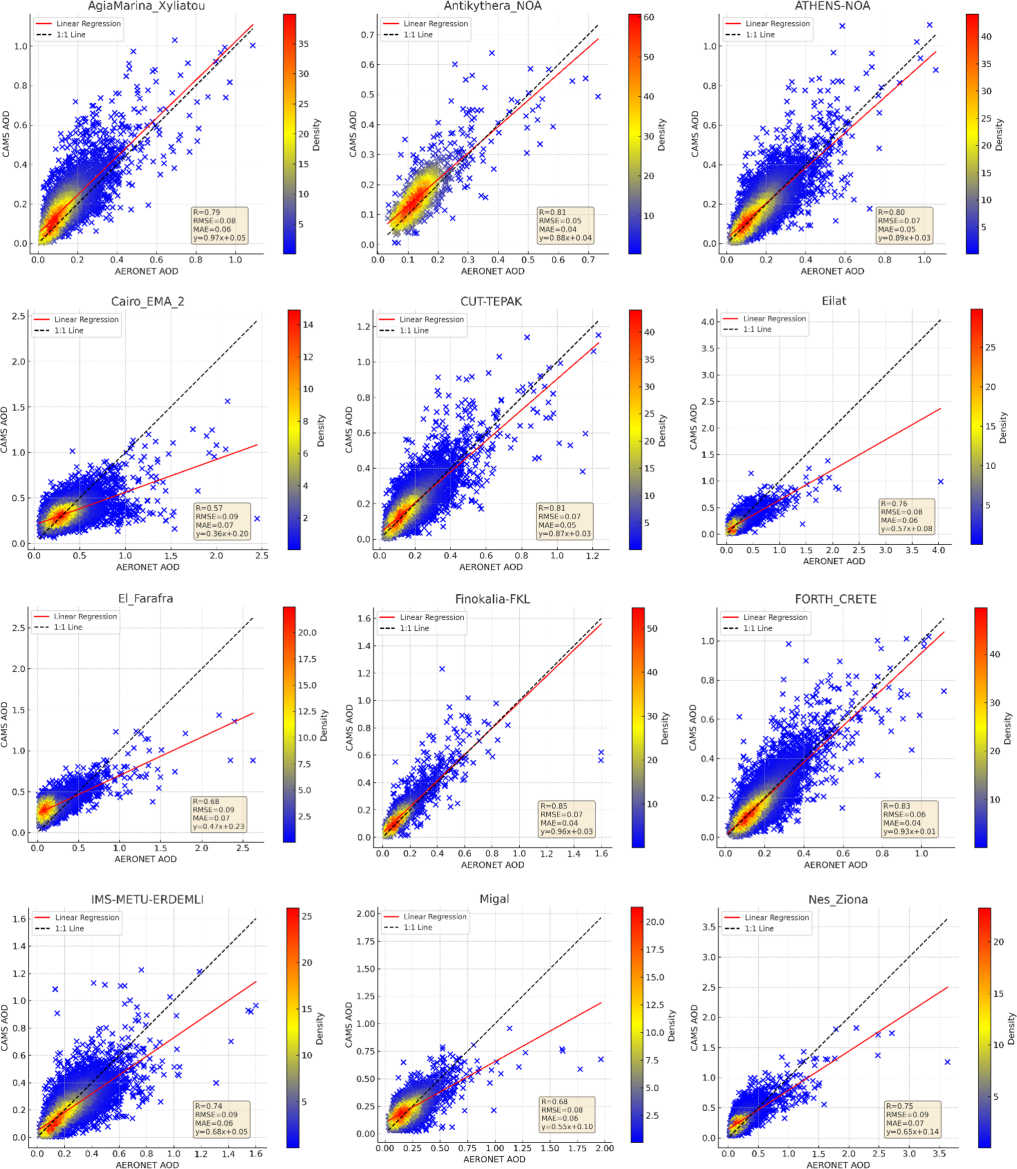

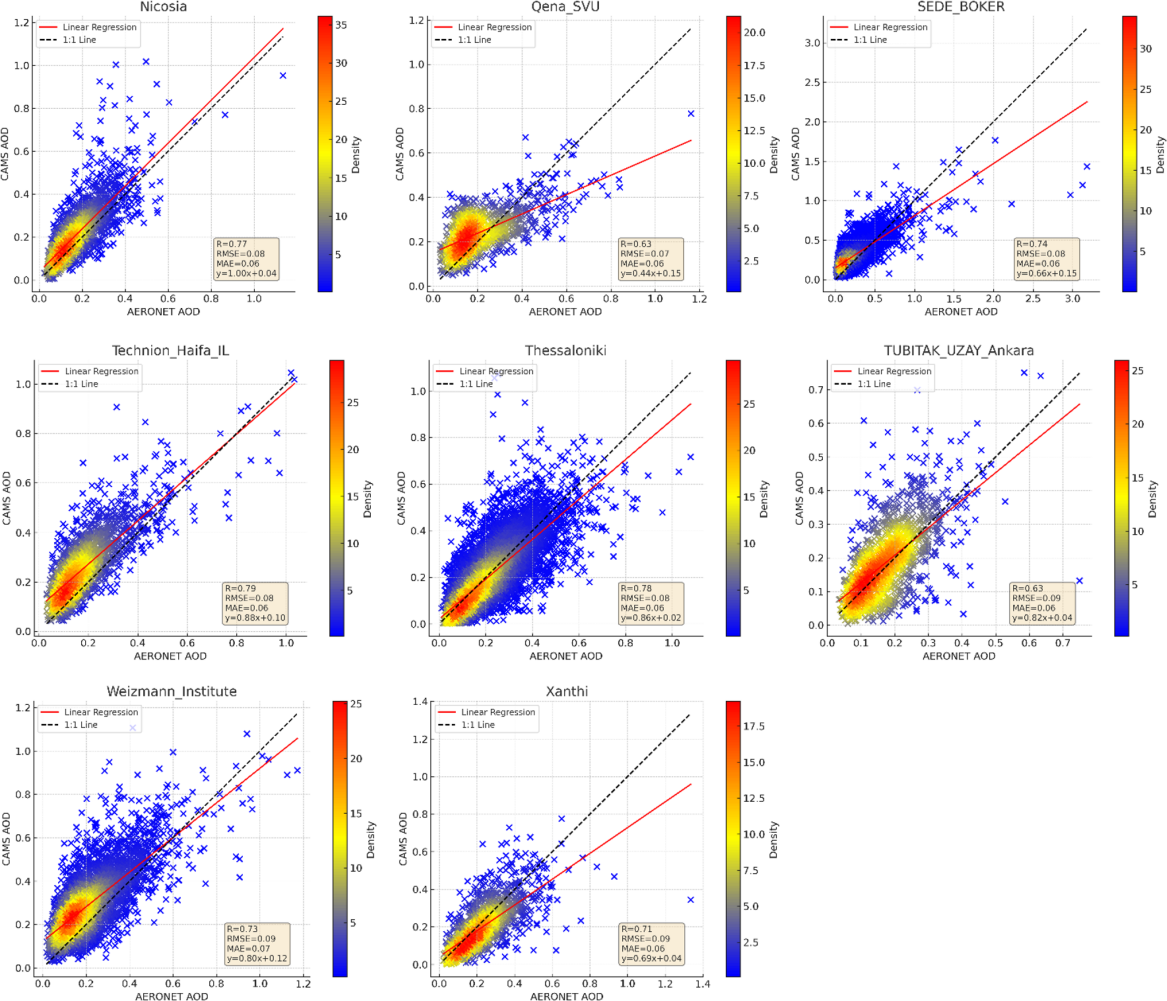
Station-based scatter plots of CAMS versus AERONET AOD measurements

Figure 3 compares CAMS AOD data against AERONET measurements for each station. Correlation coefficients ranged from 0.57 to 0.85, with correlations below 0.70 at five stations. Nevertheless, most (75%) exhibited correlations above 0.70, highlighting a good agreement. Figure 3 also demonstrates station-specific variances in the agreement between CAMS and AERONET AOD values, with some southern stations (Eliat, Migal, Nes-Ziona, SEDE-BOKER in Israel, and Cairo-EMA-2, El_Farafra, Qena_SVU in Egypt) showing a slope of the regression curve less than 1:1, indicating lower AOD values from CAMS. Challenges such as limited satellite retrievals over bright surfaces could contribute to the increased uncertainty in AOD at these locations.

For Cyprus, AERONET is consistent at all three stations, with correlations between 0.77 and 0.81. Notably, at the CUT-TEPAK station, there is a tendency for CAMS to underestimate AERONET AOD, while at AgiaMarina_Xyliatou and Nicosia, CAMS AOD is slightly higher. The discrepancies at these stations may stem from their distinct locations; for example, CUT-TEPAK’s urban setting may introduce more pollutants compared to the rural locales of the other stations (Fountoulakis et al. 2021), affecting aerosol composition and concentration.

In Turkey, similar to the CUT-TEPAK station in Cyprus, CAMS AOD underestimates AERONET observations. The correlation coefficient is higher at the coastal IMS-METU-ERDEMLI station compared to the inland TUBITAK_UZAY_Ankara station. In Egypt, the CAMS AOD data exhibit lower correlation coefficients ranging from 0.57 to 0.68, suggesting limited accuracy; however, consistency is noted at the El_Farafra and Qena_SVU stations.

**Fig. 4:**
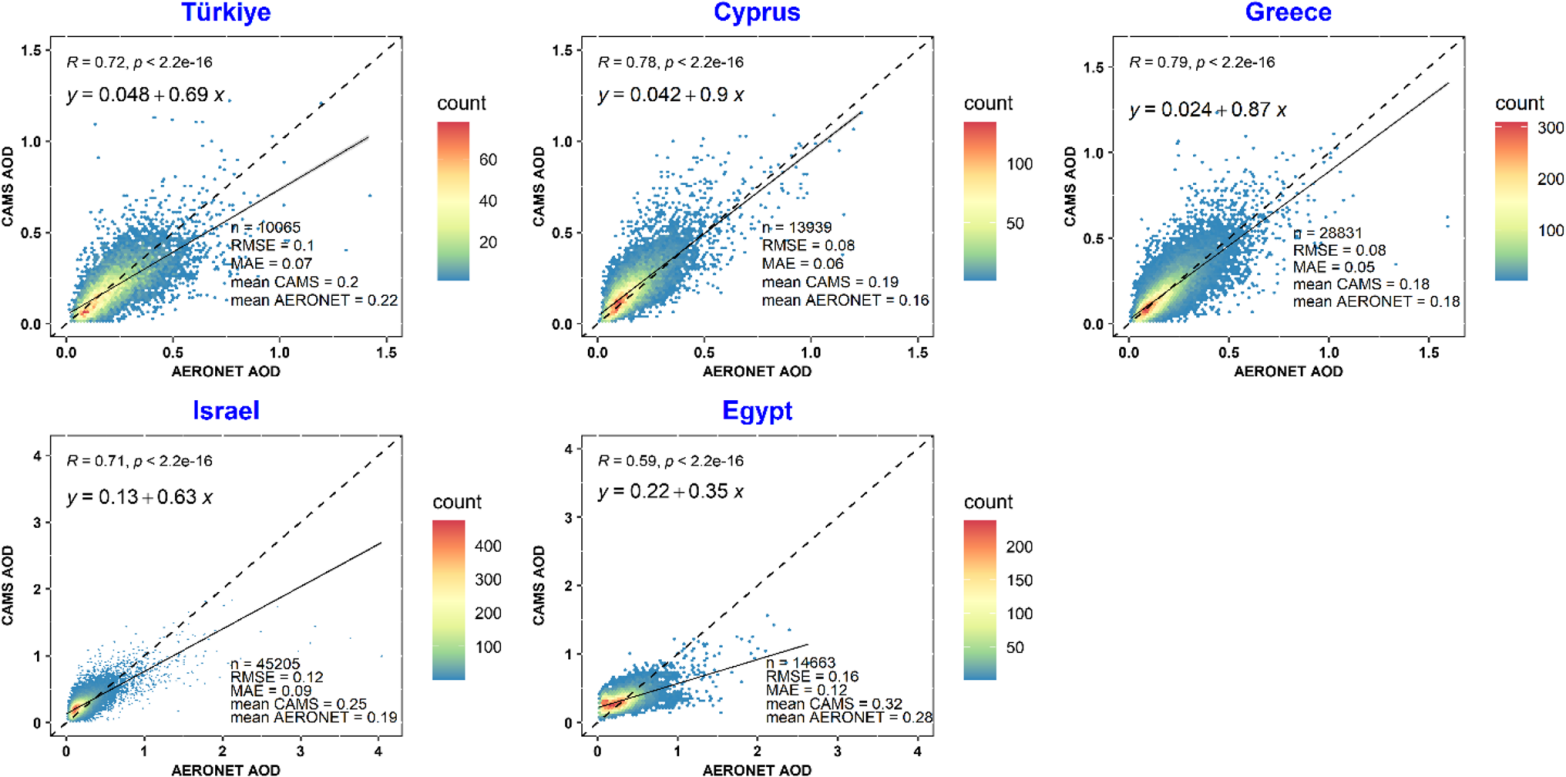
Country-based comparison of CAMS and AERONET AOD measurements

The results endorse the CAMS reanalysis products for accurate use in most Cyprus, Greece, Israel, and Turkey stations. Yet, there are indications of limited accuracy in parts of Egypt. Country-based statistical analysis, given in Fig. 4, further corroborates these findings, with performance varying notably by country. The northern countries generally display higher correlation coefficients than the southern ones, and the western regions align more closely with AERONET measurements than the eastern regions.

CAMS AOD’s lower correlation in the extensive desert regions of Egypt hints at the limitations in accurately capturing the complex aerosol dynamics in desert environments. In Israel, reasonable agreement is observed between CAMS and AERONET, except for the Migal station in the desert region, paralleling the patterns seen in Egypt’s desert areas. Additionally, the higher correlation coefficients in the northern Mediterranean may be influenced by the prevalence of forest and agricultural land, which contrasts with the southern regions’ arid and residential land use. Vegetation in the northern areas could contribute to lower AOD values due to plant leaves’ absorption and scattering of aerosols (Yang et al. 2022), thus offering a plausible explanation for the observed regional disparities.

The study scrutinizes the normalized difference vegetation index (NDVI) to discern the impact of vegetation on CAMS AOD’s correlation with AERONET data. NDVI, indicative of vegetative density, spans from − 1 to + 1, with higher figures denoting more lush vegetation. Figure S3 in the Supplementary Material showcases the Eastern Mediterranean’s NDVI spectrum, ranging from sparse in southern locales to moderate elsewhere. In areas with scant vegetation, the correlation coefficients between CAMS AOD and AERONET measurements range from 0.57 to 0.65. In contrast, regions with more substantial vegetation exhibit stronger correlations, often surpassing 0.70. This pattern suggests vegetation may bolster AOD measurement accuracy, influencing aerosol behaviors like deposition and scattering. Yet, deviations such as the lower-than-anticipated correlations at TUBITAK_UZAY_Ankara and Migal stations indicate that other factors, including urban aerosol emissions and unique atmospheric conditions, also play a pivotal role in AOD data validation.

### Diurnal and seasonal validation results

**Fig. 5:**
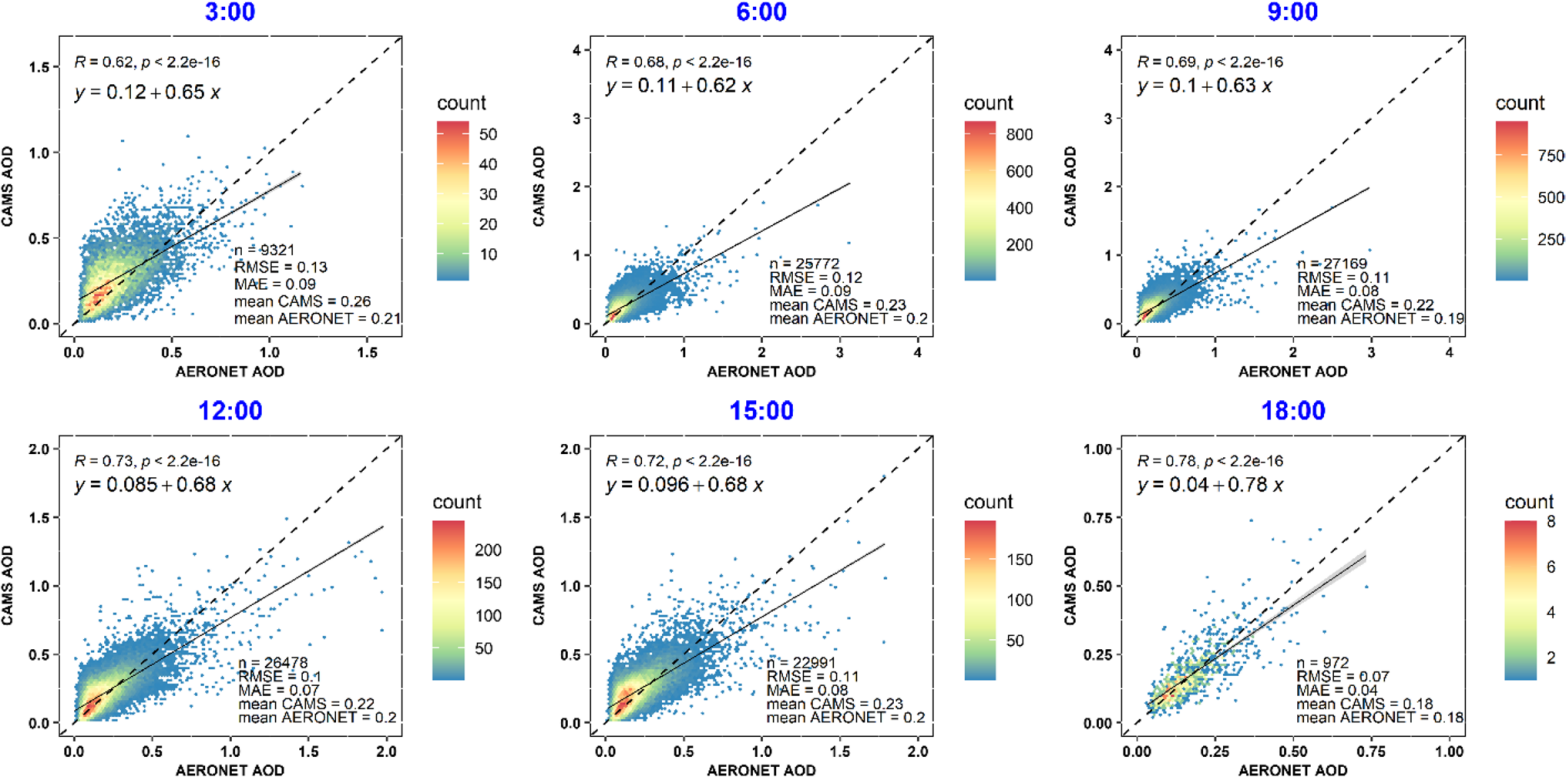
Diurnal patterns in CAMS vs. AERONET AOD validation

The diurnal variation in CAMS AOD validation against AERONET was investigated by analyzing performance metrics at various times throughout the day. With matchup counts ranging from 972 to 27,169, the correlation coefficients remain relatively high, from 0.62 to 0.77, peaking at 18:00 due to fewer data points (Fig. 5). As indicated by Zhou and Wang (2017), reanalysis data is typically assimilated from daytime satellite products, with limited data available at night. Correspondingly, RMSE and MAE values in this study exhibit a downward trend from morning to late afternoon, which is in line with findings suggesting that Aqua satellite retrievals conducted in the afternoon are more accurate than those from the Terra satellite in the morning (Bright and Gueymard 2019; Tuna Tuygun et al. 2020; Tuna Tuygun et al. 2021). Thus, CAMS AODs demonstrate commendable performance across all hours, particularly in the late afternoon. The improved performance in the late afternoon could be linked to the daily cycle of the atmospheric boundary layer height, which is known to influence aerosol mixing and distribution. The higher solar radiation during daytime and the dip in surface-level relative humidity around noon are likely to contribute to the enhanced accuracy of AOD estimations by minimizing the impact of hygroscopic growth on aerosol measurements, further elucidating the diurnal pattern observed in aerosol source variability and atmospheric conditions.

The comprehensive methodologies for determining aerosol hygroscopic growth, as outlined by Rovelli et al. (2016), alongside the observed enhancements in AOD estimation accuracy through controlled relative humidity conditions detailed by Zhang et al. (2021) and Silva et al. (2015), suggest that the precise management of surface-level relative humidity around noon, rather than solely the variations in solar radiation, plays a critical role in minimizing the hygroscopic growth impact on aerosol measurements. This refined understanding underscores the importance of accurately characterizing aerosol hygroscopic properties under varying atmospheric conditions to elucidate the nuanced diurnal patterns observed in aerosol source variability and atmospheric dynamics.

**Fig. 6:**
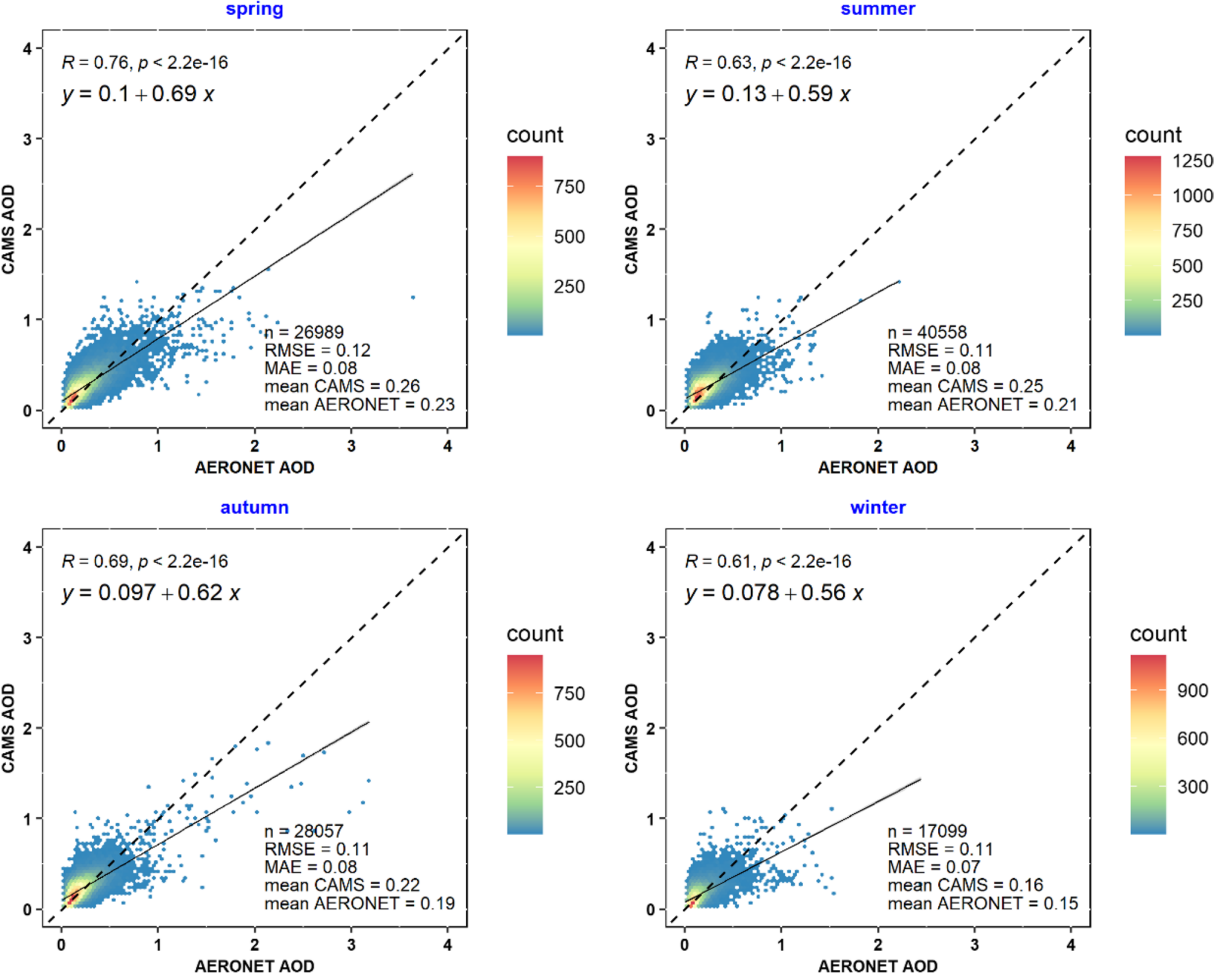
Seasonal validation results of CAMS AOD

Seasonal validation results in Fig. 6 show the strongest correlation coefficient in spring at 0.76, with the lowest in winter at 0.61. These patterns are supported by corresponding RMSE and MAE values. The CAMS AOD values tend to align well with AERONET across all seasons; however, underestimation becomes more pronounced during the summer and winter. Misra et al. (2020) also found lower correlation coefficients in winter. Challenges in obtaining accurate surface reflectance during wet conditions and the enhanced extinction efficiency of hydrophilic aerosols in high humidity could exacerbate discrepancies between satellite and ground-based AOD measurements in these seasons.

The seasonal analysis of CAMS AOD performance in the Eastern Mediterranean reveals distinct trends. Typically, higher AOD values are observed during spring, which could be associated with natural seasonal phenomena. Meteorological factors, such as variable wind speeds and temperature fluctuations, may also affect aerosol transportation and AOD levels. The summer months are characterized by warmer and drier conditions, which are conducive to the presence of different aerosol types, including those of anthropogenic origin, potentially affecting AOD measurements. Conversely, in the autumn and winter, certain natural aerosol sources are reduced, aligning with expected seasonal vegetation cycles and meteorological changes, possibly leading to lower observed AOD values. Furthermore, the influence of desert dust, particularly from northern Africa and the Middle East, is a notable factor in the seasonal AOD variability within the region, with spring and summer typically experiencing more frequent dust events. These factors combined contribute to the seasonal patterns seen in CAMS AOD data, underscoring the complex interplay of environmental and climatic influences on regional aerosol levels.

## Conclusions

This study assessed the accuracy of CAMS AOD data by leveraging 19 years of AERONET observations across the Eastern Mediterranean. The analysis revealed that while CAMS and AERONET AOD values correlate well at most stations, with coefficients frequently exceeding 0.70, notable variations exist on a regional basis. Northern countries consistently showed higher correlation coefficients than their southern counterparts, and Western regions aligned more closely with AERONET measurements than Eastern regions. Overall, CAMS tended to underestimate AOD, particularly under high aerosol conditions, aligning with findings from other studies in China, such as Song et al. (2018) and Zhang et al. (2020). The discrepancies at high AOD values are largely due to missing emissions in the CAMS aerosol model.

CAMS AOD data were more accurate in regions with low anthropogenic impact and dense vegetation. In contrast, accuracy diminished in areas with high aerosol loads, such as deserts and urban centers, and at higher AOD levels. CAMS exhibited limited accuracy in Egypt’s extensive desert regions, likely due to complex aerosol behavior. However, a reasonable agreement was observed in Israel, except for the Migal station in the Negev desert, suggesting regional similarities in desert aerosol dynamics.

Diurnal variations in CAMS performance were also evident, with better results noted in the afternoon and evening. This finding points toward potential improvements by addressing the model’s morning performance and enhancing satellite data retrievals during these hours. Seasonally, CAMS achieved a better correlation with AERONET during spring and autumn. The reduced performance in summer and winter may be due to unaccounted aerosol sources in the CAMS model.

In summary, CAMS AOD data emerged as a generally reliable tool for aerosol monitoring in the Eastern Mediterranean. However, periodic validation is recommended to refine the model, particularly during the morning and certain seasons. Policymakers and air quality managers may find these insights valuable for regional air quality assessment and management strategies. Moving forward, continuous improvement and validation efforts are essential to enhance the precision of CAMS AOD data, especially in areas with intense aerosol activities. The study’s findings also hold potential for advancing aerosol model parameterizations and deepening our understanding of aerosol-environment interactions.

### Supplementary Information

Below is the link to the electronic supplementary material.

Supplementary file1 (DOCX 1069 KB)

## Data Availability

Data will be made available on reasonable request.
