# Supplementary material for: Comparative analysis of CAMS aerosol optical depth data and AERONET observations in the Eastern Mediterranean over 19 years
Source: Environ Sci Pollut Res Int. 2024 Mar 19;31(18):27069–84. doi: 10.1007/s11356-024-32950-6 (PMC11052789; doi:10.1007/s11356-024-32950-6)
Supplement: Supplementary file 1 — Supplementary file1 (DOCX 1069 KB) [file 11356_2024_32950_MOESM1_ESM.docx]

**SUPPLEMENTARY MATERIAL**

**Comparative Analysis of CAMS Aerosol Optical Depth Data and AERONET Observations in the Eastern Mediterranean Over 19 Years**

Gizem Tuna Tuygun, Tolga Elbir

*Department of Environmental Engineering, Faculty of Engineering, Dokuz Eylul University, Buca-Izmir, Türkiye*

**List of Figures :**

**Fig. S1.** Five-year interval spatial variation of CAMS AOD in the Eastern Mediterranean

**Fig. S2.** Overall validation results in the region

**Fig. S3.** NDVI map of the study area


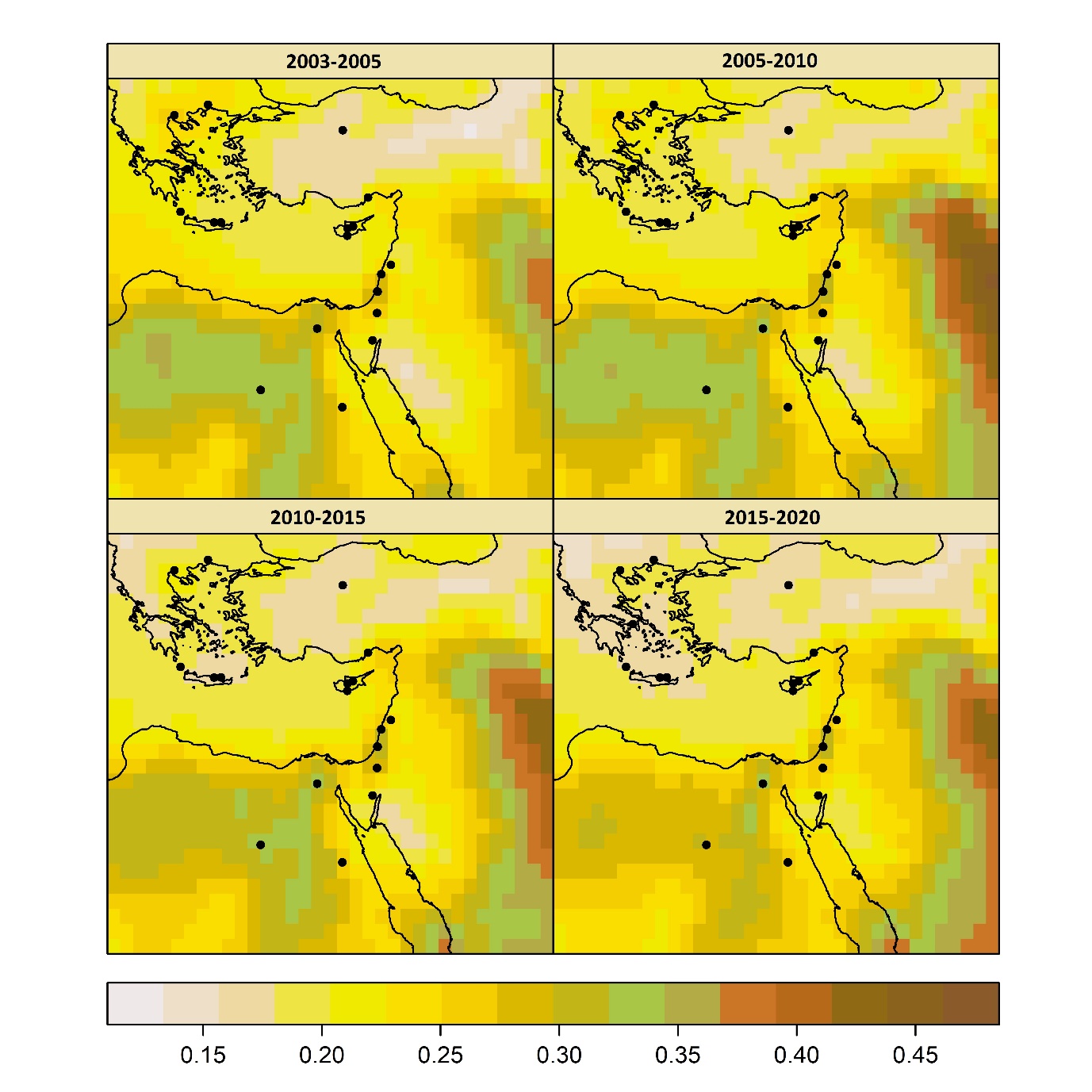


**Fig. S1.** Five-year interval spatial variation of CAMS AOD in the Eastern Mediterranean


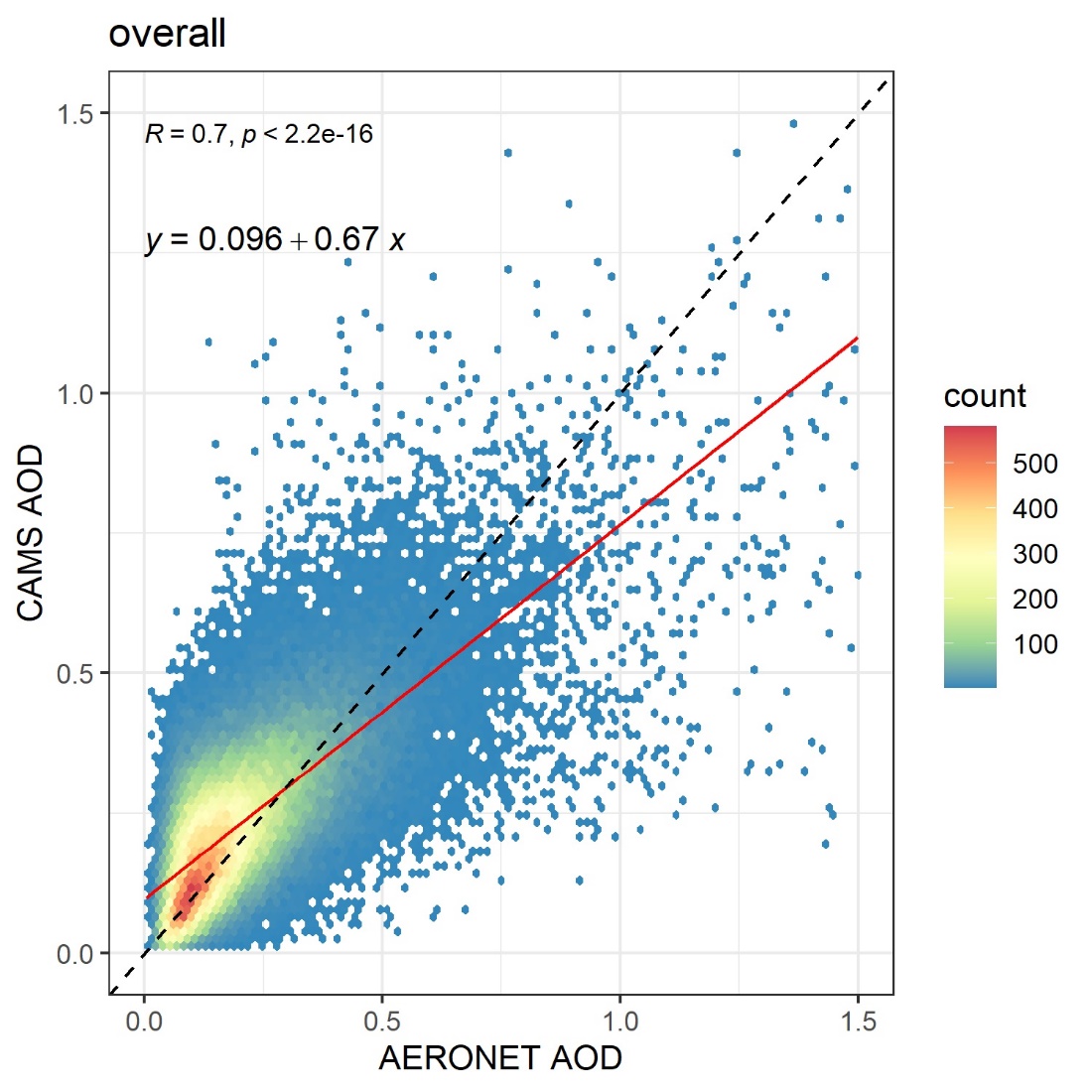


**Fig. S2.** Overall validation results in the region


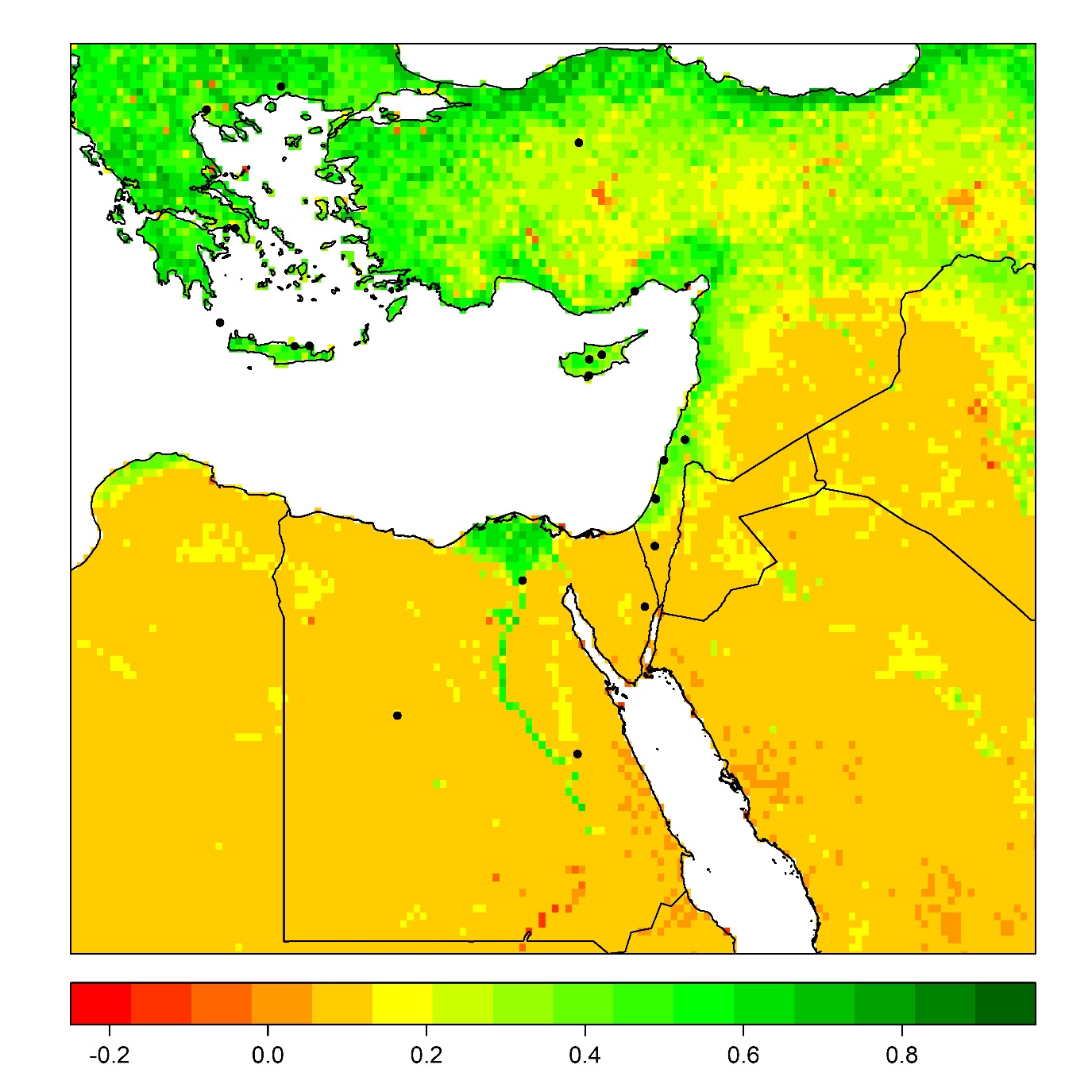


**Fig. S3.** NDVI map of the study area
